# DNA double-strand break induction in Ku80-deficient CHO cells following Boron Neutron Capture Reaction

**DOI:** 10.1186/1748-717X-6-106

**Published:** 2011-09-05

**Authors:** Yuko Kinashi, Sentaro Takahashi, Genro Kashino, Ryuichi Okayasu, Shinichiro Masunaga, Minoru Suzuki, Koji Ono

**Affiliations:** 1 Research Reactor Institute, Kyoto University, Kumatori-cho, Sennan-gun, Osaka 590-0494, Japan; 2 Oita University, Faculty of Medicine, Hazama-cho, Yufu-city, Oita 879-5593, Japan; 3 National Institute of Radiological Sciences, 4-9-1 Anagawa, Inage-ku, Chiba 263-8555, Japan

**Keywords:** xrs-5, DNA-DSB, BNCR, gamma-H2AX, 53BP1

## Abstract

**Background:**

Boron neutron capture reaction (BNCR) is based on irradiation of tumors after accumulation of boron compound. ^10^B captures neutrons and produces an alpha (^4^He) particle and a recoiled lithium nucleus (^7^Li). These particles have the characteristics of high linear energy transfer (LET) radiation and have marked biological effects. The purpose of this study is to verify that BNCR will increase cell killing and slow disappearance of repair protein-related foci to a greater extent in DNA repair-deficient cells than in wild-type cells.

**Methods:**

Chinese hamster ovary (CHO-K1) cells and a DNA double-strand break (DSB) repair deficient mutant derivative, xrs-5 (Ku80 deficient CHO mutant cells), were irradiated by thermal neutrons. The quantity of DNA-DSBs following BNCR was evaluated by measuring the phosphorylation of histone protein H2AX (gamma-H2AX) and 53BP1 foci using immunofluorescence intensity.

**Results:**

Two hours after neutron irradiation, the number of gamma-H2AX and 53BP1 foci in the CHO-K1 cells was decreased to 36.5-42.8% of the levels seen 30 min after irradiation. In contrast, two hours after irradiation, foci levels in the xrs-5 cells were 58.4-69.5% of those observed 30 min after irradiation. The number of gamma-H2AX foci in xrs-5 cells at 60-120 min after BNCT correlated with the cell killing effect of BNCR. However, in CHO-K1 cells, the RBE (relative biological effectiveness) estimated by the number of foci following BNCR was increased depending on the repair time and was not always correlated with the RBE of cytotoxicity.

**Conclusion:**

Mutant xrs-5 cells show extreme sensitivity to ionizing radiation, because xrs-5 cells lack functional Ku-protein. Our results suggest that the DNA-DSBs induced by BNCR were not well repaired in the Ku80 deficient cells. The RBE following BNCR of radio-sensitive mutant cells was not increased but was lower than that of radio-resistant cells. These results suggest that gamma-ray resistant cells have an advantage over gamma-ray sensitive cells in BNCR.

## Background

Kyoto University Research Reactor Institute (KURRI) has been investigating BNCT since 1990. BNCT has been utilized in the treatments of malignant glioma, malignant menigioma, malignant melanoma, Paget's disease, recurrent head and neck cancers, and lung tumors.

The principle underlying the Boron Neutron Capture Reaction (BNCR) is that tumor cells containing ^10^B can be destroyed efficiently by the ^10^B(n,α)^7^Li fission reaction through the delivery of effective thermal neutron doses at the target depth. During this reaction, an alpha particle and a recoiling ^7^Li ion with an average total kinetic energy of 2.34 MeV are released when compounds containing ^10^B that have accumulated in the tumor cells are exposed to thermal neutrons. These particles have the characteristics of high linear energy transfer (LET) radiation and produce enhanced biological effects. For example, it is generally accepted that high LET radiation induces more DNA-DSBs than low LET radiation.

DNA-DSBs are potentially lethal lesions created by ionizing radiation, and can be repaired by homologous recombination (HR) or non-homologous end joining (NHEJ) in mammalian cells. A number of essential proteins, including DNA-dependent protein kinase (DNA-PK), DNA-ligase IV, Rad50, and Artemis have been identified as regulators of NHEJ. Ku proteins are a component of DNA-dependent protein kinase (DNA-PK), and are involved in the repairing of DNA-DSBs by NHEJ. Xrs-5 cells (Ku80 mutant) lack functional Ku-protein, and are defective in DNA-dependent protein kinase (DNA-PK)-mediated non-homologous end-joining (D-NHEJ). Consequently, xrs-5 cells show high radiosensitivity to gamma, X-ray, or heavy-ion irradiation [1-3]

We report here that the amount of DNA damage induced by BNCR is significantly greater in D-NHEJ-defective cells compared with wild-type CHO-K1 cells, suggesting that a deficiency in the repair of DSBs indeed contributes to the enhanced sensitivity of D-NHEJ-defective cells to BNCR.

## Methods

### Cell culture

CHO K-1 (wild-type) cells and xrs5 cells (Obtained from Dr. P. Jeggo) were cultured at 37°C in a humidified 5% CO_2 _atmosphere in α-minimal essential medium (MEM) supplemented with 10% heat-inactivated calf serum (56°C for 30 min), penicillin (100 units/ml), and streptomycin (100 μg/ml). The cells were grown as a monolayer and maintained in the late exponential phase when the surface of the flask was almost confluent.

### Boron compound and neutron irradiation

A stock solution of ^10^B-para-boronophenylalanine (BPA) and B-10 enriched boric acid (1000 μg/ml) was used for all experiments. The ^10^B concentrations were measured by prompt gamma ray (PGA) spectrometry using a thermal neutron guide tube installed at KUR.

CHO K-1 cells and xrs-5 cells exponentially growing in MEM were trypsinized and cell suspensions were incubated with 25 μg/ml boric acid or BPA at 1 hour prior to the neutron irradiation. The cells were placed in to the Teflon tube and irradiated at room temperature by neutrons from the 1MW research reactor at Kyoto University.

### Radiation sources and measurement of neutron fluences

The Heavy Water Column of the Kyoto University Research Reactor was used for 1MW neutron irradiation. The thermal neutron fluences were measured by gold foil (3 mm in diameter, 0.05 mm thick) activation analysis. The gamma-ray dose including secondary gamma rays was measured with a Mg_2_SiO_4 _(Tb) thermo luminescence dosimeter. Boron concentrations in the cells were taken to be equivalent to those in the medium, as reported previously [4]. The total absorbed dose resulting from thermal or epithermal neutron irradiation was calculated by the sum of the absorbed doses, which primarily was a result of the ^1^H(n,γ)^2^D, ^14^N(n,p)^14^C, and ^10^B(n,α)^7^Li reactions according to Kobayashi's model [5]. The dose-converting coefficients of the ^1^H(n,γ)^2^D and ^14^N(n,p)^14^C reactions and details of the calculation method were described previously [5,6]. Gamma-ray irradiation was produced using a cobalt-60 gamma-ray irradiator in the Research Reactor Institute, Kyoto University at a dose rate of 1.0 Gy/min.

### Cell survival assay

**Figure 1:**
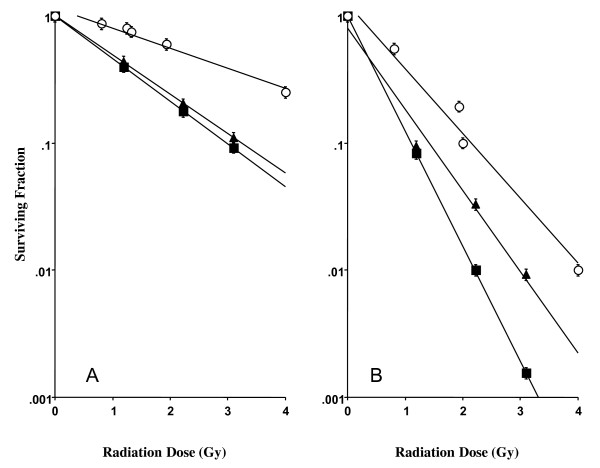
**Cell survival curves following neutron irradiation**. Survival curves are shown as a functional dose in Gy for CHO-K1(A) and xrs-5 (B) cells exposed to gamma-rays (open circle) and thermal neutrons. 25ppm of ^10^B BPA (black triangle) or boric acid (black square) was incubated on the cells for 1 hr before neutron irradiation. Each point and error bar represents the mean ± SE of three or more independent experiments.

Survival curves were obtained using standard colony formation assays. The cells were rinsed twice in PBS and suspended in fresh medium for 10-15 min after irradiation. Cells were plated in plastic Petri dishes (100 mm in diameter) at densities ranging from 100 to 10,000 cells per dish. Cells were incubated for an additional 7-10 days to allow colony formation. The cells were then fixed and stained, and colonies containing more than 50 cells were counted as survivors. Cell survival after irradiation was normalized to the survival level of un-irradiated control cells. The survival fraction was calculated and the survival curves were obtained for each cell line. The D_10 _values were derived by linear regression analysis from the survival curves (Figure 1). The RBE (relative biological effectiveness) was obtained by the ratio of mean value of D_10 _compared to that of gamma rays (Table 1).

**Table 1:** Survival parameter D_10 _doses and their RBE values calculated from dose-survival fraction curves (Fig.1) of CHO-K1 and xrs-5 cells irradiated with KUR thermal neutron beam and gamma rays.

	CHO-K1		xrs 5	
	**BPA(25ppm)**	**Boric acid** **(25ppm)**	**Boric acid** **(25ppm)**	**BPA(25ppm)**
	
D_10 _(Gy) BNCR	3.1 ± 0.2	1.1 ± 0.1	2.9 ± 0.2	1.4 ± 0.1

D_10 _(Gy) Co^60 ^gamma ray	6.2 ± 0.3		2.0 ± 0.1	

RBE^a^	2.0	1.8	2.1	1.4

^a ^RBE(relative biological effectiveness) was obtained by the ratio of mean value of D_10 _compared to that of gamma rays.

### Immunofluorescent staining of γ-H2AX and 53BP1

Cells were carefully poured on to 22 × 22 mm cover slips in 6-well micro plates filled with medium lacking boron, 30, 60, 90, and 120 min after 1Gy irradiation, the cells were fixed with 4% formaldehyde in PBS, permeabilized for 10 min on ice in 0.5% Triton X-100 in PBS, and washed thoroughly with PBS. The cover slips were then incubated with antibody-against histone H2AX phosphorylated at serine 139 (Upstate Biotechnology Inc., NY, USA) or anti-53BP1 antibody (Bethyl Laboratories, TX, USA) in TBS-DT (20 mM Tris-HCl, 137 mM NaCl, pH 7.6, containing 50 mg/ml skim milk and 0.1% Tween-20) for 2 hours at 37°C. After incubation with primary antibody, the cells were washed with PBS, and Alexa Fluor 488-labeled anti-mouse IgG and Alexa Fluor 594-labeled anti-rabbit IgG secondary antibodies (Invitrogen) were added. The cover slips were incubated for 1 hour at 37°C, washed with PBS, and sealed onto glass slides with 0.05 ml PBS containing 10% glycerol (Wako, Osaka, Japan) and 20 μg/ml DAPI (4',6-diamidino-2-phenylindole; Invitrogen, CA, USA). The cells were examined using both an Olympus fluorescence microscope (Olympus, Tokyo, Japan) and a Keyence fluorescence microscope (Keyence, Osaka, Japan), and the green intensity of the phospho-H2AX signal on digitized images was analyzed using Dynamic Cell Count (Keyence) or Adobe Photoshop version 7.0 (Adobe Systems Inc., CA, USA). The average number of foci per cell was determined in 500 cells from the three independent studies. The total area of the high intensity of green γ-H2AX and red 53 BP1signals, 30-120 min after irradiation in cell populations were determined using the Keyence software,- Dynamic Cell Count-. The RBE (relative biological effectiveness) was obtained by calculating the ratio of average number of foci per cell induced by BNCR divided by average number of foci per cell induced by gamma rays-irradiation (Tables 2, 3).

**Table 2:** The average number of foci per cell and their RBE^a ^values in CHO-K1 cells irradiated by the KUR thermal neutron beam.

Time after irradiation & Boron compound		BNCR				Gamma-ray	
		
		Average number of γ-H2AX foci/cell	RBE	Average number of 53BP1 foci/cell	RBE	Average number of γ-H2AX foci/cell	Average number of 53BP1 foci/cell
30 min	BPA	16.7 ± 4.2	0.85	15.6 ± 3.0	1.10	19.7 ± 3.0	14.2 ± 2.0
			
	Boric Acid	19.2 ± 4.0	0.97	16.8 ± 3.0	1.18		

60 min	BPA	10.8 ± 4.0	1.26	8.8 ± 3.0	1.16	8.6 ± 3.0	7.6 ± 2.0
			
	Boric Acid	14.0 ± 4.2	1.63	10.4 ± 3.0	1.37		

90 min	BPA	9.0 ± 3.2	1.50	8.0 ± 2.1*	2.67	6.0 ± 2.2	3.0 ± 1.2
			
	Boric Acid	11.0 ± 3.0	1.83	9.2 ± 3.0*	3.07		

120 min	BPA	7.4 ± 3.0*	2.64	6.4 ± 1.0*	6.40	2.8 ± 2.0	1.0 ± 0.9
			
	Boric Acid	7.0 ± 3.2*	2.50	7.2 ± 1.1*	7.20		

^a ^RBE(relative biological effectiveness) was obtained by the ratio of average number of foci per cell compared to that of gamma rays.

*Significantly higher (p < 0.05) compared to gamma-ray irradiation.

**Table 3:** The average number of foci per cell and their RBE^a ^values in xrs-5 cells irradiated by the KUR thermal neutron beam.

Time after irradiation & Boron compound		BNCR				Gamma-ray	
		
		Average number of γ-H2AX foci	RBE	Average number of 53BP1 foci	RBE	Average number of γ-H2AX foci	Average number of 53BP1 foci
30 min	BPA	24.8 ± 4.0	1.07	22.3 ± 4.1	1.16	23.1 ± 3.0	19.2 ± 2.0
			
	Boric Acid	26.0 ± 4.2	1.13	24.0 ± 4.0	1.25		

60 min	BPA	22.6 ± 4.0*	1.86	20.4 ± 4.2*	1.73	12.1 ± 3.0	11.8 ± 2.0
			
	Boric Acid	24.7 ± 4.2*	2.04	22.8 ± 4.0*	1.93		

90 min	BPA	18.4 ± 3.2*	1.67	19.4 ± 3.1*	1.90	11.0 ± 2.2	10.2 ± 1.2
			
	Boric Acid	20.2 ± 3.0*	1.84	21.6 ± 3.0*	2.12		

120 min	BPA	15.6 ± 2.0*	1.73	15.5 ± 2.0*	1.76	9.0 ± 2.0	8.8 ± 1.0
			
	Boric Acid	15.2 ± 2.1*	1.69	16.6 ± 2.0*	1.89		

^a ^RBE(relative biological effectiveness) was obtained by the ratio of average number of foci per cell compared to that of gamma rays.

* Significantly higher (p < 0.05) compared to gamma-ray irradiation

Statistical significance was calculated using the Student's *t-*tests. Results were considered significant for p values < 0.05.

## Results

The survival fractions of the CHO-K1 cells and the xrs-5 cells exposed to thermal neutrons and gamma rays are shown in Figure 1A and 1B. The level of cell survival following neutron irradiation decreased exponentially without a shoulder in the survival curve. The D_10 _dose parameters for survival following neutron and gamma-ray irradiation and their RBEs are listed in Table 1.

**Figure 2:**
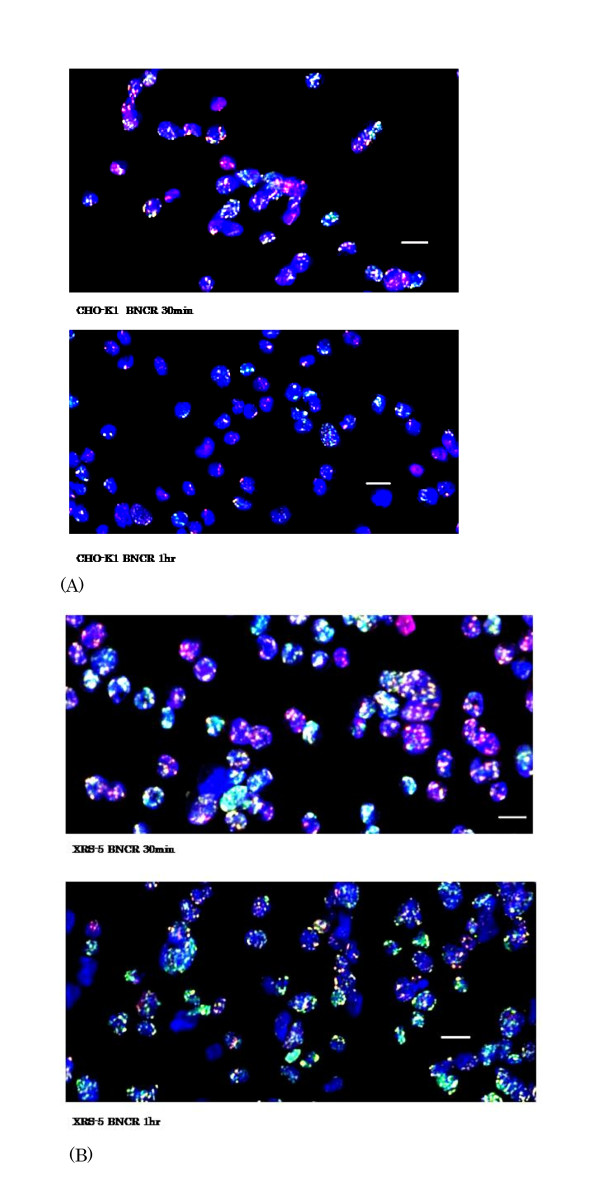
**Representative multi color images of nuclear γ-H2AX and 53BP1 foci in the CHO-K1 (A) and xrs-5 (B) cells at 30 and 60 min after the BNCR with 25ppm BPA**. Nuclei were stained with DAPI (blue). γ-H2AX (green) and 53BP1 (red) foci are shown in the same photo. The bar represents 20 μm.

Figure 2 shows multi scan photos of the immunofluorescent staining of γ-H2AX and 53BP1 foci in the CHO-K1 (Figure 2-A) and xrs-5 (Figure 2-B) cells after 1Gy of the BNCR and gamma irradiation. The time-dependent variation of the average number of γ-H2AX and 53BP1 foci per cell up to 2 hours post-irradiation is shown in Table 2 and 3. In the CHO-K1 cells, the number of γ-H2AX and 53BP1foci was reduced 2 hours after thermal neutron irradiation. However, in the xrs-5 cells, 58.4-69.5% of the foci in the xrs-5 cells observed at 30 min after the irradiation were still remained at two hours post-irradiation. These results indicate that the xrs-5 cells have not repaired their DNA-DSBs 2 hours after BNCR.

**Figure 3:**
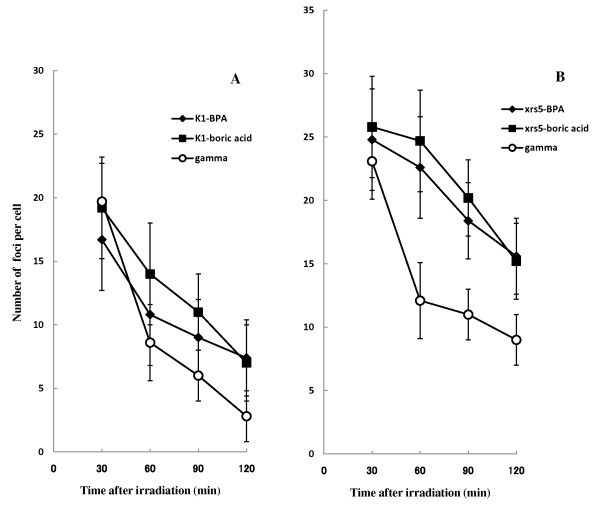
**Induction and loss of induced nuclear γ-H2AX foci in CHO-K1 (A) and xrs-5 cells (B) determined up to 120 min after 1Gy of thermal neutron or gamma irradiation**. In the BNCR study, 25 ppm of BPA (black diamond) and boric acid (black square) was used. The data represent the average number ± SE of γ-H2AX foci per cell in three or more independent experiments.

Figure 3 shows the time dependent loss of γ-H2AX foci following 1Gy of neutron and gamma-ray irradiation in the CHO-K1 cells (Figure 3-A) and xrs-5 cells (Figure 3-B). The number of γ-H2AX foci per cell 30 min post irradiation was significantly higher in xrs-5 cells compared with CHO-K1 cells for BNCR and gamma-ray irradiation. In the case of CHO-K1 cells, the DNA-DSBs in CHO-K1 cells following gamma-ray irradiation were well repaired, so the number of γ-H2AX foci 120 min post gamma-ray irradiation was lower than that observed after BNCR treatment. For the xrs-5 cells, the DNA-DSBs in xrs-5 cells following gamma-ray irradiation were also better repaired than following BNCR. However, the number of γ-H2AX foci per cell 120 min post irradiation was significantly higher in xrs-5 cells compared with CHO-K1 cells for BNCR and gamma-ray irradiation.

Tables 2 and 3 show the BNCR-RBE value as the ratio of average number of γ-H2AX and 53 BP1 foci per cell compared to that of gamma rays. The RBE values estimated by DNA-DSB focus assay varied depending on the assay time following BNCR irradiation.

**Figure 4:**
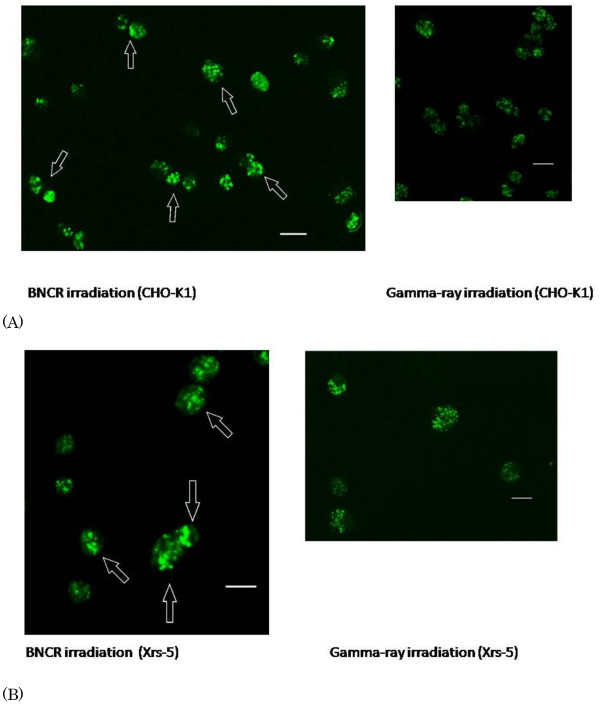
**Representative images of nuclear γ-H2AX foci in the CHO-K1 (A) and xrs-5 (B) cells at 30 min after 2Gy of BNCR with 25ppm BPA compared with 1Gy gamma irradiation**. Many large foci were observed in the cells exposed BNCR (open arrows). The bar represents 20 μm.

**Figure 5:**
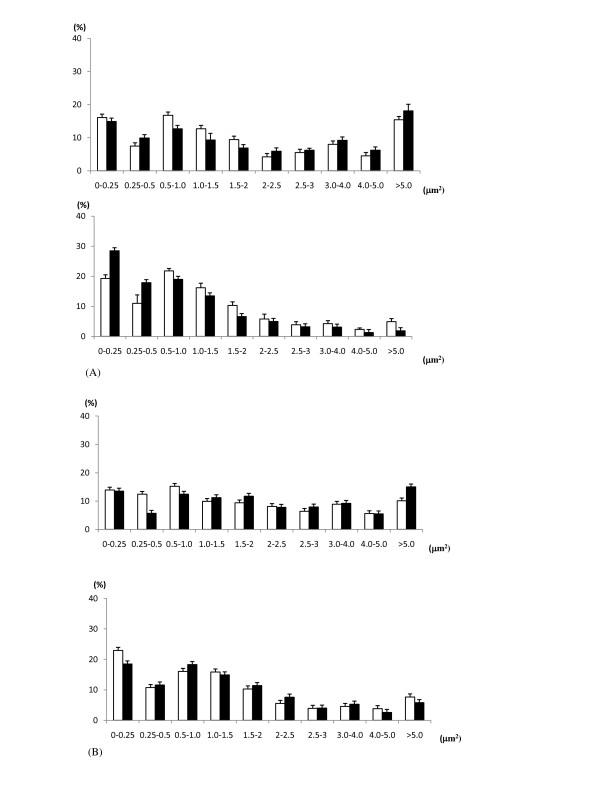
**The histograms of γ-H2AX foci size at 30 min (white bar) and 120 min (black bar) after 1Gy in CHO-K1 (A) and xrs-5 cells (B)**. The upper graph shows the BNCR study and the lower graph shows the gamma-irradiation study. Each bar shows the average ± SE of three independent experiments.

Figure 4 shows photos of γ-H2AX foci in the CHO-K1 (Figure 4-A) and xrs-5 (Figure 4-B) cells after 1Gy of the BNCR and gamma irradiation. Here, we found that the mean area size of γ-H2AX foci induced by BNCR were significant larger than those by gamma-rays. Figure 5 shows the distribution histograms of the focus area size measured using the BZ-9000 BZII image analysis system (KEYENCE). BNCR induced larger γ-H2AX foci than that observed after gamma-ray irradiation in the both cell lines. The size distribution of γ-H2AX foci 120 min post irradiation was not different with 30 min post gamma-ray irradiation, while on the other hand, the size distribution of γ-H2AX foci 120 min post irradiation was larger compared with 30 min post BNCR irradiation.

## Discussion

We employed CHO-K1 cells and a DNA double-strand break (DSB) repair deficient derivative line (xrs-5) to investigate the relationship between DNA-DSB damage and cytotoxic potential following BNCR. In BNCR, it is thought that alpha particles and lithium atoms (which have a traveling range of 10-14 μm) from the ^10^B(n,α)^7^Li fission reaction cause DNA-DSBs. NHEJ-deficient xrs-5 cells (Ku80 mutant) are markedly sensitive to cell killing by gamma- or X-ray irradiation [1,2]. In this report, we have shown that DNA-DSB repair deficient cells are also more sensitive to BNCR compared with low LET (linear energy transfer) irradiation. However, the RBE of the gamma-ray sensitive xrs-5 cells was not larger than that of the gamma-ray resistant CHO-K1 cells. In a previous study of BNCR, we reported that the RBEs of radio-resistant cells were larger than those of radio-sensitive cells using various malignant glioma cell lines [7]. These results suggested that the gamma-ray resistant cells have an advantage over the gamma-ray sensitive cells in BNCR.

**Figure 6:**
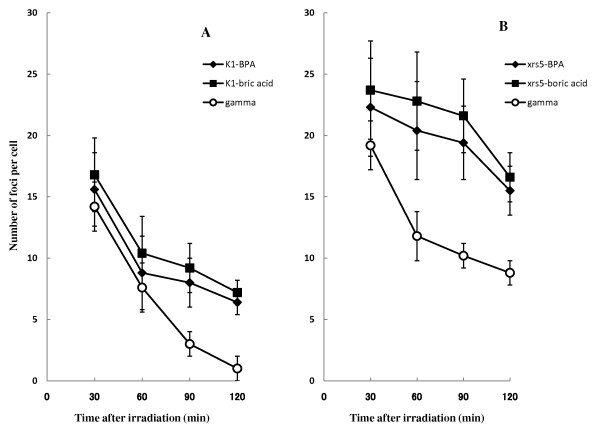
**Induction and loss of induced nuclear 53BP1 foci in CHO-K1 (A) and xrs-5 cells (B) determined up to 120 min after 1Gy of thermal neutron or gamma irradiation**. In the BNCR study, 25 ppm of BPA (black diamond) and boric acid (black square) was used. The data represent the average number ± SE of 53BP1 foci per cell in three or more independent experiments.

We investigated the induction and persistence of DNA damage using DNA-DSB signals. The numbers of γ-H2AX and 53BP1 foci induced by BNCR were significantly greater in xrs-5 compared with CHO-K1 cells (Figures 3, 6). Figure 3 shows that the initial number of γ-H2AX foci induced per cell following BNCR was not greatly increased compared with gamma-ray irradiation. During the period following the BNCR reaction, many unrepaired DNA-DSBs remained in the xrs-5 cells (Figures 3, 6). Using the CHO-K1 and xrs-5 cell lines, it was previously reported that differences in repair capacity depended greatly on high LET, heavy-ion induced damage, consistent with our results [3].

The initial step of the cellular response to DNA-DSBs is the phosphorylation of histone H2AX, which induces γ-H2AX foci [8,9]. Subsequently, the 53BP1 protein accumulates at DSB lesions with γ-H2AX [10]. It has been reported that γ-H2AX foci are a particularly sensitive DNA damage marker following low-dose irradiation [11]. In our study, there was no variation in the appearance of γ-H2AX or 53BP1 foci in the CHO-K1 cells. In the xrs-5 cells, 53BP1 foci were greatly induced by BNCR relative to gamma-rays. H2AX foci were increased to a lesser extent by BNCR (Figures 3-B,6-B). We calculated the BNCR-RBE value as the ratio of average number of foci per cell compared to that of gamma rays (Tables 2, 3). The BNCR-RBEs estimated using the number of γ-H2AX foci in CHO-K1 and xrs-5 cells were 0.85-2.50 and 1.07-2.04, respectively. The BNCR-RBEs estimated using the number of 53BP1 foci in CHO-K1 and xrs-5 cells were 1.10-7.20 and 1.16-2.12, respectively.

In the case of CHO-K1 cells, the RBEs estimated by foci number following BNCR was varied and increased depending on the repair time. The BNCR-RBEs at 60, 90, and 120 min after BNCR in xrs-5 cells were substantially consistent and correlated with the cell killing effect of BNCR. The DSBs remaining after BNCR irradiation suggested that the constant RBE values were due to the inability of xrs-5 cells to carry out DNA repair.

In survival studies using mammalian cells, as the LET increases, the RBE increases slowly at about 10 keV/μm and more rapidly reaches a maximum at about 100 keV/μm [12]. Using a chromosomal damage assay induced by heavy ions, Kawata et al. reported that the RBE for chomatid breaks reached a maximum value of around 2 at a LET of about 100 keV/μm [13]. In BNCR studies, the LET range of the 1.47 MeV (α-particle) and 0.84 MeV (^7^Li-particle) particles are 50-231 keV/μm and 65-266 keV/μm, respectively. Using DSB repair-deficient cells, other researchers reported that the RBE did not increase with high LET irradiation [14-16]. Our RBEs for xrs-5 cells were not greater than the RBEs for wild-type CHO-K1 cells and are consistent with other reports concerning high LET and NHEJ-deficient cells. Previous studies showed that RBE correlated with the repair capacity of the cells, and the RBE of repair-deficient cells was smaller than that of repair-proficient cells [17]. These studies reported that a RBE maximum was found at LET values between 150 and 200 keV/μm in a repair-proficient cell (CHO-K1 cell) line; on the other hand, in a repair-deficient cell line (xrs-5), the RBE failed to show a maximum, and decreased continuously for LET values above 100 keV/μm, using high LET carbon ions of different energies [17].

Previous studies of the induction and rejoining of DNA-DBSs in mammalian cells exposed to high LET radiation demonstrated that the number of γ-H2AX foci did not always correlate with cell killing [18]. In a study of melanoma cells irradiated with proton and lithium beams, it was reported that the size of γ-H2AX foci was an accurate parameter correlating the rejoining of DSBs induced by different types of LET radiation and radio sensitivity [19]. In our BNCR study, the size of γ-H2AX foci induced by neutron irradiation was unequal and rather larger than the size of foci induced by gamma rays (Figures 4, 5). It was previously reported that irradiation with 130 keV/μm high LET, nucleon nitrogen ions increased the size of induced γ-H2AX foci, while the size of low LET, x-ray induced γ-H2AX foci did not change [20]. Another study reported that the size of γ-H2AX foci was increased after high LET lithium beam irradiation, and was correlated with cell killing [19]. Here, we revealed that BNCR induced larger γ-H2AX foci, depending on time after irradiation, while on the other hand, the BNCR-induced foci in the CHO-K1 and xrs5 cells consisted of mixed small and large foci. The thermal neutron beam from KUR is a wide range beam that includes gamma rays and prompt gamma rays. At the absorbed dose of 2Gy, the composition of ^4^He and ^7^Li ions, thermal neutrons, epithermal neutrons, fast neutrons, and gamma rays are 63.1, 4.6, 0.5, 3.4, and 28.4%, respectively. These various components of the absorbed dose explained the lower RBE and lower DNA-DSB damage. In addition, the distribution of boron in the nucleus of the cells and the bystander effect of BNCR [21] will affect the efficiency of high LET radiation on cell survival and DNA-DSBs.

## Conclusions

Our study demonstrated that BNCR induced high cytotoxic effects and low capacity to repair DNA-DSBs in NHEJ repair-deficient mutant xrs-5 cells. The RBE following BNCR of radio-sensitive mutant cells was not increased and rather was lower than that of radio-resistant cells. These results suggest that gamma-ray resistant cells have an advantage over gamma-ray sensitive cells in BNCR.

## Competing interests

The authors declare that they have no competing interests.

## Authors' contributions

YK conceived of the study, participated in the design of the study and carried out the immunoassays. ST participated in the design of the study. GK carried out the immunoassays. RO participated in the design of the study. SM participated in the design of the study. MS performed the statistical analysis. KO participated in the design of the study. All authors read and approved the final manuscript.
